# The use of percutaneous-endoscopic rendezvous stenting in a patient with bile duct injury after cholecystectomy—and a unique complication requiring secondary endoscopic intervention

**DOI:** 10.1093/jscr/rjab119

**Published:** 2021-04-20

**Authors:** Anna A Vidovszky, Fred Qafiti, S James El Haddi, Theodore Doukides, Nir Hus, Thomas Genuit

**Affiliations:** Florida Atlantic University Charles E. Schmidt College of Medicine, Boca Raton, FL, USA; Department of Surgery, Florida Atlantic University Charles E. Schmidt College of Medicine, Boca Raton, FL, USA; Department of Surgery, Florida Atlantic University Charles E. Schmidt College of Medicine, Boca Raton, FL, USA; Florida Atlantic University Charles E. Schmidt College of Medicine, Boca Raton, FL, USA; Department of Gastroenterology, Division of Advanced Endoscopy, Delray Medical Center, Delray Beach, FL, USA; Department of Surgery, Florida Atlantic University Charles E. Schmidt College of Medicine, Boca Raton, FL, USA; Department of Surgery, Delray Medical Center, Delray Beach, FL, USA; Department of Surgery, Florida Atlantic University Charles E. Schmidt College of Medicine, Boca Raton, FL, USA

## Abstract

Bile duct injury (BDI) is a potentially devastating complication after cholecystectomy. Familiarity with the diagnosis and multidisciplinary treatment options is imperative. This report highlights the utility of the rendezvous stenting procedure in a high-risk patient and describes a rare complication involving stent misplacement through the surgical drain. This is a 96-year-old female patient who suffered a Strasburg Class D injury during cholecystectomy, repaired over a T-tube. The T-tube dislodged postoperatively. Endoscopic and transhepatic stenting attempts were unsuccessful. Ultimately, a rendezvous approach allowed successful deployment of a covered metal stent. The stent was inadvertently deployed through a side fenestration of a surgical drain and was explanted upon drain removal. Repeat endoscopic stent placement was successful. The patient recovered without further complication. Surgical drains near the BDI can become sources of unexpected complications. A higher index of suspicion and careful interpretation of procedural imaging studies may prevent this complication.

## INTRODUCTION

Laparoscopic cholecystectomy is the standard of care in treatment of acute cholecystitis. Laparoscopic surgery may be more prone to bile duct injury (BDI) than open repair (0.2–1.5% versus 0.1–0.2%, respectively) [1]. In most laparoscopic cholecystectomies, BDI’s were recognized intraoperatively (72.9%), with the remainder either identified within 30-days of surgery (13.8%) or between 31 and 365 days (14.3%) [2].

BDI can be managed nonoperatively by endoscopic or percutaneous transhepatic biliary stenting. These modalities independently are often successful. Anatomic distortion from the extent of disease may require a combined approach. An endoscopic-transhepatic rendezvous approach is successful in 80.4% of patients [3]. Common complications include acute pancreatitis, acute cholangitis and peritonitis (totaling 4.2%), followed by major bleeding (3.6%), pulmonary complications (2.4%), anesthesia-related complications (1.5%) and liver biloma (0.5%) [3].

## CASE REPORT

A 96-year-old female patient presented to the emergency room with a 4-day history of abdominal pain and diarrhea. Her past medical history included bovine aortic valve replacement and hyperlipidemia. The patient presented afebrile, with abdominal distension and right upper quadrant (RUQ) tenderness to palpation. Laboratory studies demonstrated leukocytosis to 17 800 cells/mcL, alkaline phosphatase of 135 IU/L, with otherwise normal liver enzymes. Computed tomography (CT) of the abdomen with intravenous contrast demonstrated a distended gallbladder with no wall thickening, pericholecystic fluid or biliary ductal dilation. Hepatobiliary iminodiacetic acid scan demonstrated absence of gallbladder filling, adequate liver uptake and excretion of contrast into the duodenum, confirming acute cholecystitis. The patient began to hemodynamically decompensate and was taken for urgent laparoscopic cholecystectomy.

**Table 1:** Relevant case studies demonstrating rendezvous intervention and related complications

Study	Age/Sex	Primary diagnosis	Indication	Intervention	Complications
Kimura *et al.* [4]	64/F	Wilson disease – living donor liver transplant with right lobe graft and hepaticojejunostomy	Obstructive cholangitis hepaticojejunostomy due to donor stones at anastomosis	Lithotomy performed via rendezvous using double balloon endoscope	Intrahepatic stones in branches of intrahepatic ducts required second lithotomy
La Barba *et al.* [5]	Series of 200 patients	Cholecysto-choledocholithiasis	Symptomatic cholecysto-choledocholithiasis	Laparoscopic cholecystectomy with intraoperative cholangiogram, transcystic guidewire insertion into distal CBD, endoscopic rendezvous snare with introduction of sphincterotome over wire	Pancreatitis (3%) Pneumonia (4%) Bile leak (2%) Abscess (1%) Bleeding (3%) Other (1.5%)
Gkekas *et al.* [6]	41/F	Cholelithiasis	Intraoperative diagnosis of choledocholithiasis	Laparoscopic cholecystectomy with intraoperative cholangiogram, transcystic guidewire insertion into distal CBD, endoscopic rendezvous snare	Omentum and stomach strangulation via trans-cystic guidewire; conversion to laparotomy

**Figure 1:**
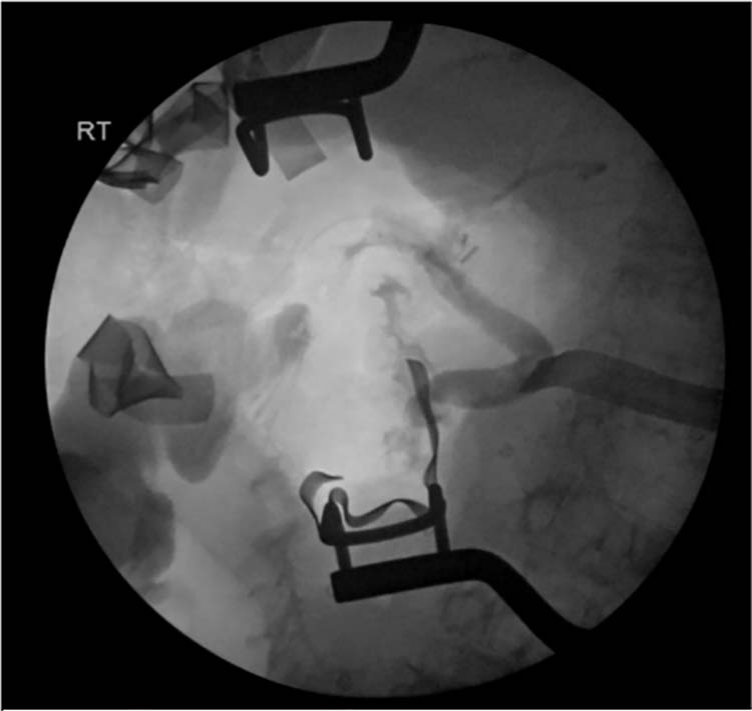
Intraoperative cholangiogram demonstrating Strasburg Type D BDI [1].

Intraoperatively, a distended, friable, gangrenous gallbladder was encountered, requiring conversion to open procedure. A Strasburg Type D partial laceration to the common bile duct (CBD) was confirmed with intraoperative cholangiogram and repaired primarily over a T-tube. A Jackson-Pratt (JP) drain was placed near the repair (Fig. 1).

The patient was ultimately discharged and lost to routine follow-up. On postoperative week eight, the patient returned to the hospital with fever and RUQ pain 24 hours after inadvertently explanting her T-tube. Magnetic resonance cholangiopancreatography demonstrated an intact biliary tree with no fluid collections. Serum bilirubin was within normal limits throughout admission, and the patient was discharged after resolution of symptoms with intravenous antibiotics.

On 2-week in-clinic follow up, the patient presented with persistent bilious drainage at the T-tube site. Endoscopic retrograde cholangiopancreatography (ERCP) and stenting with a 4 mm by 10 mm covered metal stent in the CBD was performed. The percutaneous biliary drainage reduced drastically, and the patient was discharged in anticipation of JP drain removal in clinic.

On postoperative week 12, the patient returned with persistent RUQ abdominal pain and bilious output from the JP drain. Repeat CT imaging demonstrated CBD stent migration into the pancreatic head. ERCP was performed and the stent was removed; however, the CBD could not be cannulated. Percutaneous transhepatic cholangiography was attempted and demonstrated extravasation of contrast from the lateral CBD contained by the JP drain; the distal CBD could not be visualized. An 8Fr external biliary drain was left within the common hepatic duct.

**Figure 2:**
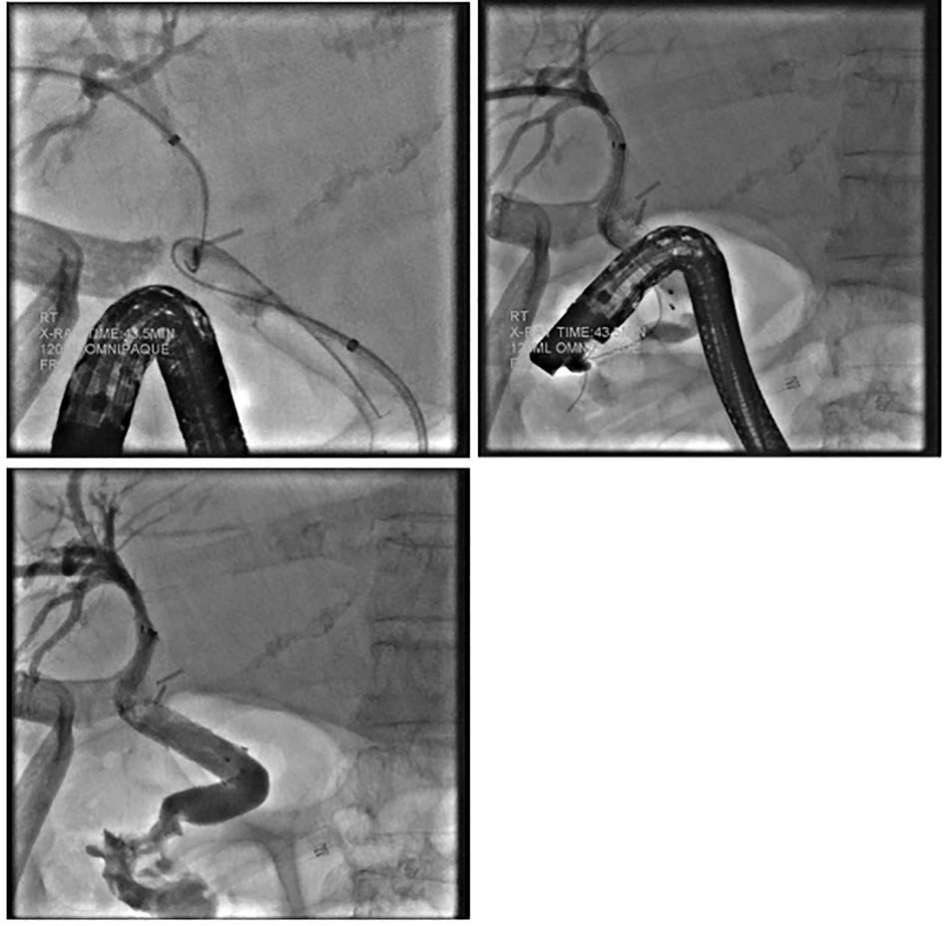
ERCP demonstrating successful rendezvous with deployment of metal stent in the vicinity of surgical drain.

Despite external drainage, the patient experienced persistent bilious output from the JP drain. A rendezvous procedure was performed; cholangiography visualized the dilated bilateral intrahepatic ducts with narrowing at the proximal CBD. Via a simultaneous endoscopic approach, the distal CBD was successfully cannulated and balloon dilated. A 10 x 60 mm covered fluency (BARD) stent was deployed. Completion cholangiogram demonstrated contrast flowing to the duodenum with no extravasation. The patient recovered well and was subsequently discharged (Fig. 2).

**Figure 3:**
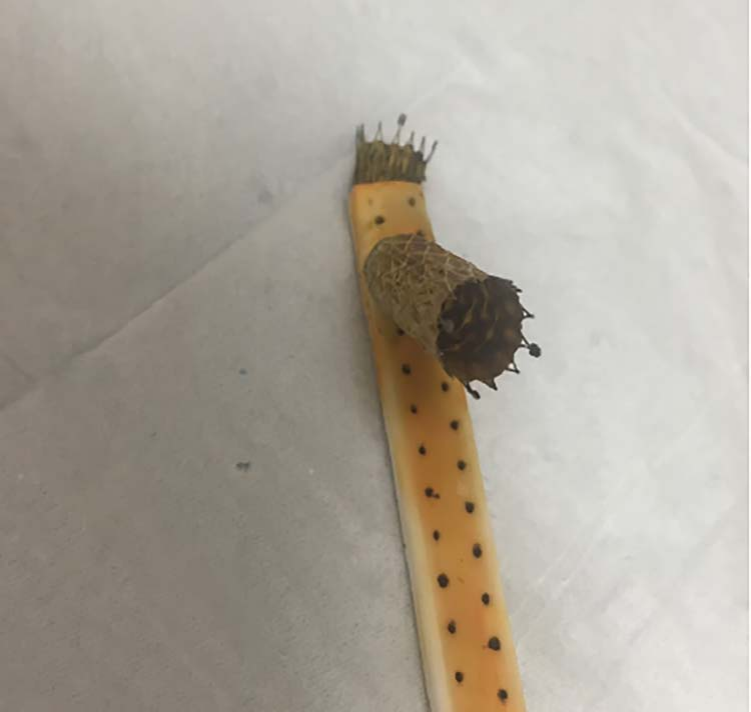
Explanted metal biliary stent deployed through surgical drain lumen.

On 5-day follow-up, the JP drainage was nonbilious and the drain was removed. On removal, the metal stent was also explanted. The stent was found to have been inadvertently deployed through a side fenestration of the JP drain lumen (Fig. 3).

**Figure 4:**
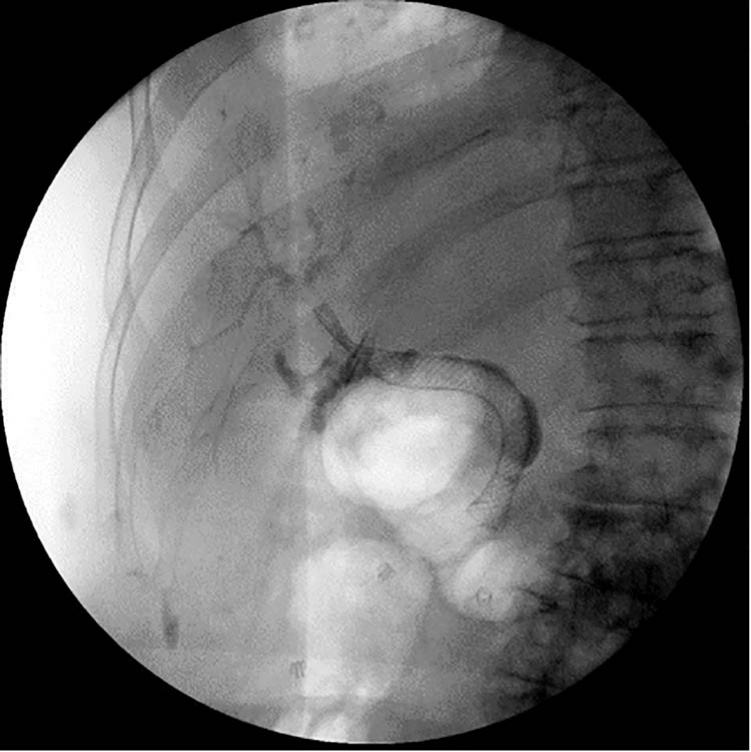
ERCP demonstrating successful replacement of metal stent after explantation.

Repeat ERCP was performed and the CBD was easily traversed. Cholangiography demonstrated bile leakage within a well-developed sinus tract. A 10 x 80 mm fully covered metal Wallflex (Boston Scientific) stent was placed (Fig. 4).

One year later, prior to scheduled stent removal, the patient’s stent had occluded, and was removed and replaced with a plastic 7Fr x 10 cm duodenal bend Advanix stent (Boston Scientific). Otherwise, no additional biliary complications were noted for the following 3 years.

## DISCUSSION

We present the resolution of a complex case involving extremity of age, severe disease, innovative intervention and a unique complication not described in literature (Table 1).

Rates of BDI from laparoscopic cholecystectomy have decreased significantly over past decades [2]. However, it remains imperative that the surgeon is well-versed in the range of techniques used to address and repair BDI.

The first reported case of a combined duodenoscopic and transhepatic approach was described almost 40 years ago, where Mason *et al.* [7] described the successful balloon dilation of a stenosed papilla. Six years later, Sommer *et al.* [8] reported rendezvous success in 21 out of 22 cases.

Decades later, the rendezvous procedure has advanced tremendously. One study analyzed the incidence of post-ERCP complications and found that there was an 8% rate of overall complication, and a 0.43% rate in mortality in 900 patients over 17 years [9]. In contrast, Isayama *et al.* [10] reported that the success rate of the more complex rendezvous procedure was 74% in 273 cases.

Since these highlights of the rendezvous procedure’s achievements, many other studies have found success in the procedure. Table 1 summarizes several reports of rendezvous application with related complications.

This interdisciplinary approach has shown promise in hepatobiliary disease management. That said, it remains paramount to involve the endoscopist and interventional radiologist early in the process.

In the case of our patient, the most unanticipated complication during this procedure was placement of the guidewire through the surgical drain. Guidewire manipulation is often the cause of failed rendezvous cannulation, and some studies have suggested using a variety of hydrophilic wires to overcome this. In order to improve visualization of the guidewire, studies have also seen success using an endoscopic-ultrasonography-guided procedure. This patient’s complex case prompts discussion on the range of methods available to address bile duct injuries, and potentially spur ideas for innovative techniques.
